# A Simple Model for the Influence of Meiotic Conversion Tracts on GC Content

**DOI:** 10.1371/journal.pone.0016109

**Published:** 2011-01-13

**Authors:** Marie-Claude Marsolier-Kergoat

**Affiliations:** Institut de Biologie et de Technologies de Saclay, CEA/Saclay, Gif-sur-Yvette, France; University College Dublin, Ireland

## Abstract

A strong correlation between GC content and recombination rate is observed in many eukaryotes, which is thought to be due to conversion events linked to the repair of meiotic double-strand breaks. In several organisms, the length of conversion tracts has been shown to decrease exponentially with increasing distance from the sites of meiotic double-strand breaks. I show here that this behavior leads to a simple analytical model for the evolution and the equilibrium state of the GC content of sequences devoid of meiotic double-strand break sites. In the yeast *Saccharomyces cerevisiae*, meiotic double-strand breaks are practically excluded from protein-coding sequences. A good fit was observed between the predictions of the model and the variations of the average GC content of the third codon position (GC3) of *S. cerevisiae* genes. Moreover, recombination parameters that can be extracted by fitting the data to the model coincide with experimentally determined values. These results thus indicate that meiotic recombination plays an important part in determining the fluctuations of GC content in yeast coding sequences. The model also accounted for the different patterns of GC variations observed in the genes of *Candida* species that exhibit a variety of sexual lifestyles, and hence a wide range of meiotic recombination rates. Finally, the variations of the average GC3 content of human and chicken coding sequences could also be fitted by the model. These results suggest the existence of a widespread pattern of GC variation in eukaryotic genes due to meiotic recombination, which would imply the generality of two features of meiotic recombination: its association with GC-biased gene conversion and the quasi-exclusion of meiotic double-strand breaks from coding sequences. Moreover, the model points out to specific constraints on protein fragments encoded by exon terminal sequences, which are the most affected by the GC bias.

## Introduction

**Figure 1:**
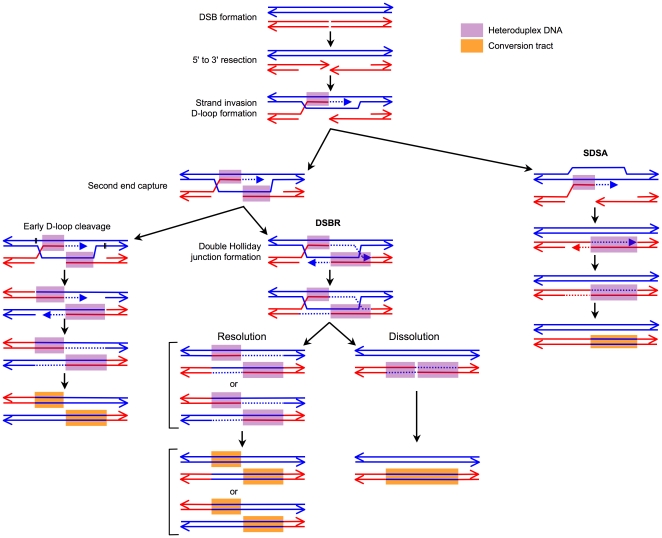
Current model of the pathways involved in meiotic DSB repair. Two interacting, homologous chromatids (out of four) are represented. Following DSB formation, 5′ to 3′ resection leads to 3′ ssDNA tails. One of these tails invades the homologous DNA, forming a D-loop which is then extended by DNA synthesis (dotted line). If the second end of the DSB is captured, either early D-loop cleavage (indicated by black segments) leads to crossover products, or, in the DSBR (Double Strand Break Repair) pathway, a double Holliday junction is formed, whose resolution or dissolution generate either crossovers or noncrossovers. Alternatively, in the SDSA (Synthesis-Dependent Strand-Annealing) pathway, the D-loop is disassembled by displacement of the newly synthesized strand, which results in noncrossovers. Heteroduplex DNA structures are present at many steps in all DSB repair pathways. Mismatch repair of heteroduplexes can probably occur at different steps, but is represented here as taking place at the last step and as generating conversion tracts, which appear to be the most frequent outcome.

Almost ubiquitous among eukaryotic organisms is a correlation between GC content and meiotic recombination rates [1]–[5]. Whereas the causality relationships are debated in many cases, several lines of evidence have accumulated for a mechanism termed GC-biased gene conversion whereby the frequency of meiotic recombination affects the evolution of GC content (for a review, see [6]). This mechanism relies on the fact that during meiotic recombination, double-strand breaks (DSBs) are repaired through a process involving the formation of DNA heteroduplexes between the strands of the cut and the uncut chromosomes (see [7], [8] for reviews). As shown in Figure 1, these DNA heteroduplexes, which are systematically formed at the sites of DSBs, can extend to variable distances away from it on both sides.

If the sequences of the strands forming an heteroduplex are not perfectly complementary, mismatches occur that can be repaired by several pathways, probably at multiple steps during the process of DSB repair. Assuming that one strand is consistently used as a template for the correction of the other strand, correcting the sequence of the cut chromosome according to the sequence of the uncut chromosome (this event is called gene conversion), leads to three copies of the sequence from the uncut chromosome and only one copy of the sequence from the cut chromosome in the recombination products, instead of the original two copies for each sequence. When comparing the sequences of the meiotic products, this asymmetry appears as a so-called conversion tract (Figure 1). In contrast, correcting the sequence of the uncut chromosome according to the sequence of the cut chromosome (this event is called gene restoration) preserves the symmetry, and both sequences remain present in two copies in the recombination products. One could imagine complex patterns with mismatch repair alternating between conversion and restoration events. However, the fact that crossovers are frequently associated with simple, continuous conversion tracts indicates that in the majority of the cases the sequence of the cut chromosome is systematically converted [9].

For a given meiosis, gene conversion introduces an asymmetry in the number of allelic sequences present in the meiotic products. If DSBs at a given site occur with the same frequency in two pairs (A and B) of homologous chromosomes, this asymmetry disappears at the level of the population, and allelic frequencies are not modified. In contrast, if DSBs occur more frequently at a given site in one of the pairs of homologs (let's say the A pair), the frequency of the sequences of the A chromosomes close to this DSB site is decreased by meiotic events, which lowers the probability of their fixation in the population.

Regarding the evolution of the GC content, a higher probability of AT-rich alleles to experience meiotic DSBs can thus lead to an increase of the GC content in the sequences surrounding DSB sites. This recombination-initiation bias is one of the models proposed for GC-biased gene conversion. Other mechanisms are possible, including biases in the repair enzymes [3], which would favor GC in cases of AT/GC mismatches. Whatever the molecular mechanism(s) ultimately responsible for it, the fact that gene conversion linked to meiotic recombination increases GC content was recently given a direct demonstration in *Saccharomyces cerevisiae* by Mancera *et al.* who measured a significant 1.4 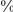 increase in the GC content of converted sequences by genotyping 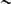 52,000 markers in all the products of 51 meioses [9].

In most cases, the frequency of gene conversion on both sides of a DSB site decreases exponentially with the distance from the DSB site [10], [11]. This can be explained by assuming that processes leading to gene conversion extend from the DSB site with a fixed probability 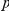 of stopping at each base pair. Let 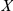 be the random variable corresponding to the distance over which a conversion tract extends in one direction from a DSB site. Experimental observations thus indicate that 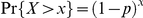. The majority of repair events leading to crossovers should involve the extension of conversion tracts on both sides of a DSB (Figure 1). Under the hypothesis that the distances over which conversion tracts extend on both sides of a DSB correspond to independent variables, the sum of the lengths of the diverging conversion tracts (hereafter called the total length of conversion tracts) has a mean value of 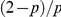 [10]. Applying this model to the experimental observations obtained by Mancera *et al.*, who found a median value of 2 kilobases (kb) for the total length of conversion tracts associated with crossovers, results in an estimate of 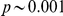 [9].

Combining the exponential decrease of conversion tract extension with the influence of these tracts on GC content, I reasoned that DNA sequences devoid of meiotic DSB sites that experience the extension of meiotic conversion tracts initiated beyond their boundaries should present a specific profile of GC content, with a higher GC content at both ends. Here I present a simple, analytical model for the evolution of the GC content of such sequences. Its predictions are consistent with the genomic data of *S. cerevisiae* and of both sexual and presumably asexual species of *Candida* and related yeasts. Finally, the model also seems to provide a relevant description for the gene sequences of higher eukaryotes, which suggests that a universal pattern of GC variation could be induced in eukaryotic protein-coding sequences by meiotic conversion tracts.

## Results

### A model for the evolution of the GC content in DNA sequences devoid of meiotic DSB sites

**Figure 2:**
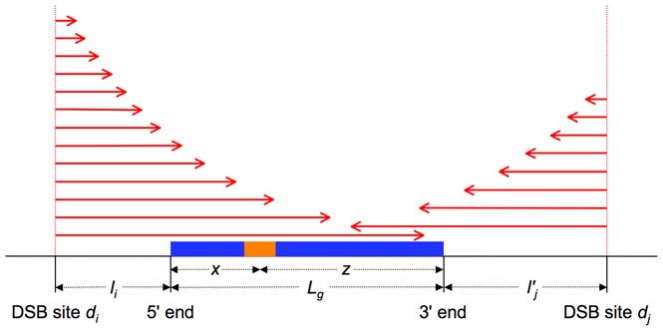
Schema illustrating some of the model parameters. A sequence 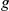 of length 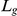, devoid of meiotic DSB sites, is shown as a blue rectangle. The orange rectangle corresponds to a segment whose middle point is located at position 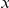 relative to the 5′ end of 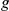 and at position 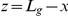 relative to its 3′ end. The red arrows represent the extension towards 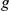 of different conversion tracts initiated either at the DSB site 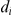, located at the distance 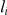 upstream of 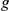 or at the DSB site 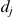, located at the distance 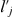 downstream of 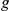.

Let's consider a segment whose middle point is located at position 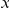 (in bp) relative to the 5′ end of a DNA sequence 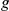 (Figure 2). The GC content of this segment evolves through the appearance and the fixation of new alleles. We will suppose that this process involves two kinds of mechanisms: (i) mechanisms dependent on meiotic recombination, which modify the probability of fixation of an allele through gene conversion and (ii) mechanisms independent of meiotic recombination, which operate uniformly on the segments of the sequence, independently of their positions 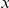.

These two kinds of mechanisms are characterized by the rates of the substitutions they induce in the genome: let 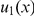 and 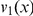 represent, respectively, the AT to GC and GC to AT substitution rates linked to recombination-dependent processes, and 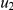 and 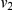 represent the substitution rates linked to recombination-independent processes, which we will consider as independent of 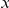.

Let 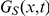 and 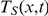 be, respectively, the GC content (proportion of GCs) of a segment located at position 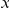, and the frequency with which conversion tracts reach 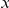 in the sequence 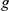 at time 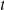. 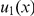 and 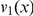 are obviously dependent on 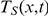. We will suppose that 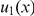 and 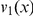 can be considered as proportional to 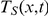 and can be written as 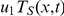 and 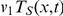, respectively, with 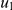 and 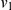 being two constants. We thus have 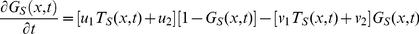
(1)


Let's calculate the frequency 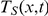. We assume that the sequences under study are devoid of DSB sites, which means that the conversion tracts affecting them originate from DSB sites located either in their 5′ or in their 3′ regions. Let's first consider a DSB site 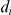 located at a distance 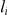 upstream of the 5′ end of the sequence 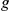, from which conversion tracts are initiated with a frequency 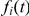 (Figure 2). 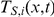, the frequency with which conversion tracts extend from DSBs at 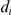 to position 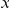, corresponds to the product of 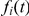 and the probability that the conversion tracts, once initiated, will extend up to 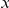. Since conversion tracts extend from DSB sites with a fixed probability 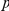 to stop at each base pair, this probability is equal to 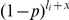. We thus get 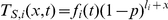.

We consider now all the DSB sites located either upstream of the sequence 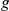, at distances 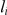 from its 5′ end, or downstream of 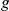 at distances 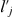 from its 3′ end, *i.e.* at distance 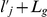 from the 5′ end, 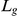 being the length of the sequence 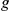 (Figure 2). The position of the DSB sites is unknown, so we simply consider all positions upstream and downstream of the sequence 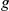 as potential DSB sites at which conversion tracts are initiated at frequencies 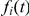, with 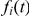 being negligible for most of them. We thus obtain 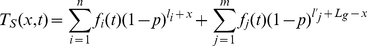
(2)with 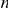 and 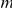 corresponding to the distances between the 5′ (respectively, 3′) end of the sequence 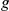 and the 5′ (respectively, 3′) end of the chromosome on which 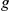 is located.

Let's consider the situation in which the frequencies 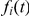 in Equation 2 can be replaced by the constants 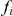 (in the Discussion, we will see that 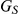 can closely approach an equilibrium value only if the frequencies 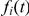 are either constant over time or undergo only rapid changes around a constant, time-averaged value 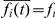 at frequencies too high to be reflected by the GC content).

For a given sequence 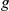, we can write 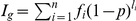 and 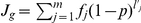, so that 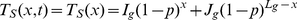
(3)



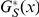
, the equilibrium value of 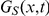, is then obtained by setting 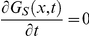 and can be written 
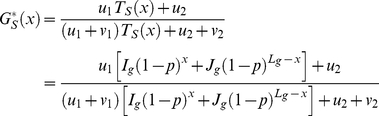
(4)


The GC content of the sequences of some organisms at least appears to be close to equilibrium (see below), hence the relevance of 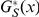.

Finally, Equation 4 can be simplified into 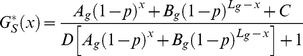
(5)with 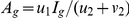 and 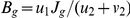 being specific to the sequence 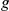 (depending on the position and on the time-averaged activity of the neighboring DSB sites) and 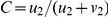 and 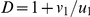 being two constants *a priori* identical for all sequences.

### Analysis of *Saccharomyces cerevisiae* coding sequences

The model makes many simplifying assumptions (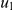, 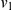, 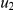, 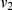 and 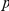 are considered to be constant over time and identical for all sequences for example) and Equation 5 applies only to specific cases, to sequences with low meiotic DSB density and whose GC contents are close to equilibrium, in organisms exhibiting GC-biased gene conversion. I first sought to evaluate its relevance by analyzing the sequences of *S. cerevisiae*, an organism in which meiotic recombination has been extensively studied and for which the existence of GC-biased gene conversion has been experimentally demonstrated [9].

Several studies have shown that the protein-coding sequences of *S. cerevisiae* experience few meiotic DSBs. A thorough mapping of meiotic DSBs on *S. cerevisiae* chromosome III at a resolution of 100–500 pb identified only 5 DSB sites in protein-coding sequences out of 76 DSB regions [12]. DSBs were also found to be practically excluded from intergenic regions containing two terminators. These results were subsequently confirmed by a genome-wide mapping of meiotic recombination hotspots [1]. Chromatin structure seems to be the most basic determinant controlling the position of meiotic DSBs in yeast (reviewed in [13]) and the preferential localization of DSB sites in promoter regions is often explained by the hypothesis that DSB-forming complexes are more efficient on open chromatin regions, which are most commonly established for promoting transcription.

The third codon position was selected for analysis because this position is the least constrained by coding requirements. To test whether the GC content of the third codon position (GC3) in *S. cerevisiae* genes was close to equilibrium, the equilibrium GC3 contents 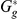 of 3661 genes of *S. cerevisiae* were computed from the inferred substitutions having occurred in *S. cerevisiae* lineage after the divergence between *S. cerevisiae* and *Saccharomyces paradoxus* (see Methods). A strong linear relationship was found between 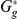 and the observed GC3 content 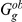 of *S. cerevisiae* genes (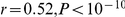): 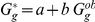 with 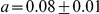 and 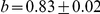. I therefore considered that the conditions were met for Equation 5 to describe approximately the GC3 content of *S. cerevisiae* protein-coding sequences.

The sequences of 5500 ORFs without intron, annotated as verified or uncharacterized in the Saccharomyces Genome Database were divided into non-overlapping segments of 66 codons (198 bp), starting either from the ATG or from the stop codon. The orientation of the coding sequences was taken into account because of the asymmetry in the distribution of the DSB sites, which are preferentially located in promoter-containing intergenes.

Equation 5 can be taken as describing the theoretical equilibrium GC3 content 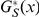 of a gene segment located at position 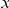 relative to the ATG. Let 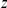 correspond to the distance between a segment and the stop codon of the gene (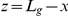, Figure 2). The theoretical equilibrium GC3 content of a gene segment can also be written 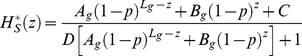
(6)


**Figure 3:**
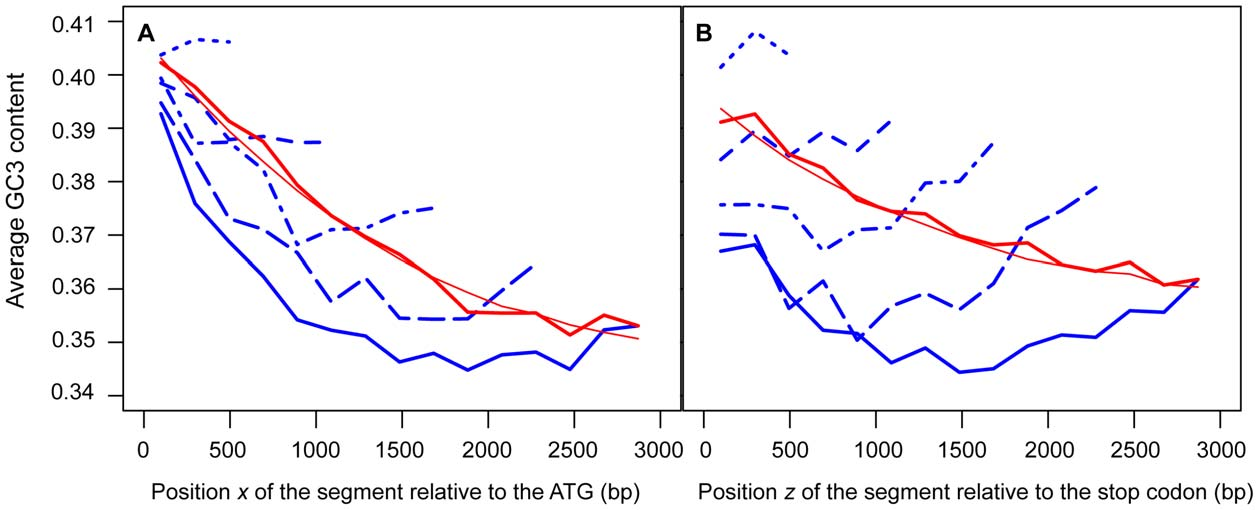
Variations of GC3 content in *S. cerevisiae* protein-coding sequences. The mean GC3 contents 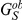 (A) or 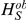 (B) of 66-codon segments are plotted as a function of the positions 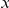 or 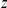 relative to the ATG or to the stop codon, respectively, on which the segments are centered. For a given position 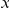 or 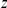, 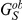 and 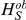 are averaged over classes of genes binned by their lengths. The genes of set 1 (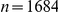, blue dotted line) contain 3 to 5 66-codon segments, the genes of set 2 (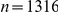, blue dashed line) contain 6 to 8 segments, the genes of set 3 (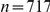, blue dot-dash line) contain 9 to 11 segments, the genes of set 4 (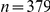, blue long-dash line) contain 12 to 14 segments, and the genes of set 5 (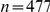, blue solid line) contain at least 15 segments. Only the average values for the segments common to all the genes of a given set are plotted (*i.e.* only the segments corresponding to the shortest genes of the set). The thick, red, solid lines represent the mean 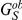 or 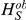 averaged over all genes containing at least one 66-codon segment (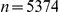). The thin, red, solid lines represent the mean theoretical values 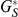 or 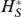 averaged over all genes containing at least one 66-codon segment and whose values of 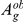 and 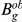 could be determined (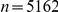). 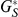 and 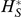 were calculated as functions of 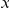, 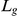, 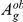 and 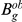 using Equations 7 and 8 (and the estimates of 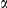, 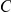, 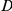 and 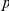 given in Table 1), and averaged over segments with the same position 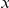 or 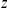, respectively.

According to the model, the shape of the curves representing 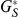 and 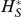 depends on 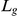 as the difference in GC3 content between the middle and the end segments of a gene increases with gene length, the middle segments of the longest genes rarely experiencing the extension of conversion tracts. The genes were therefore binned into classes according to the number of 198 bp-segments they contain. The average observed GC3 contents 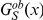 and 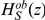 of each segment centered on position 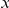 from the ATG or on position 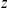 from the stop codon, respectively, were measured for the different classes of genes. As shown in Figure 3A, 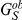 tends to decrease for segments located farther from the ends of the genes. This trend could be observed for all classes of genes, except for the shortest genes, and was most apparent for the longest genes. A similar trend was also visible for 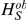 (Figure 3B).

Even if current recombination rates vary largely from one gene to another [1], [9], [14], [15], suggesting a similar heterogeneity for 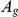 and 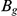, the potential gene-to-gene variations in 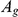 and 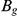 were disregarded in a first analysis, and uniform values of 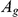, 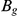, 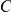, 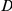 and 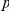 that give the best fit to the observed GC3 contents 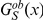 or 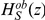 were determined (Table 1). Fitting 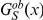 and 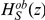 gave similar results. The fact that the estimates of 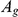 (which reflects the activity of DSB sites at the gene 5′ ends) were higher than the estimates of 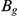 (which reflects the activity of DSB sites at the gene 3′ ends) was consistent with the observation that in current *S. cerevisiae* strains DSBs occur preferentially in promoter-containing regions and are almost excluded from intergenes containing two terminators. Similarly, the estimates of 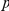, the probability of the conversion tracts to stop at each base pair, were 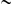 0.0009, in good agreement with the value of 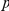 (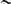 0.001) determined experimentally from the analysis of 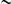 4200 crossovers in *S. cerevisiae* [9]. The Pearson's correlation coefficient between the observed GC3 contents 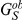 and the theoretical GC3 content 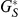 that can be calculated from Equation 5 using the estimates of 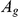, 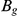, 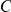, 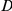 and 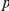 given in Table 1, was found equal to 0.25 (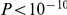, 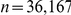).

**Table 1:** Equation coefficients for *S. cerevisiae* genes.

	Fit with 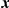 and 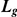		Fit with 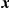 , 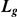 , 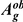 and 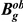	
	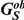	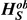	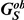	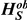
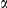	NA	NA	0.030 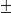 0.002	0.026 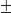 0.001
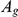	0.12 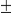 0.03	0.12 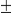 0.04	NA	NA
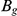	0.09 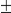 0.02	0.08 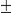 0.02	NA	NA
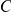	0.321 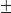 0.004	0.324 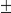 0.003	0.330 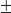 0.001	0.331 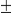 0.001
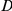	1.0 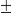 0.4	1.0 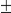 0.4	1.40 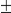 0.04	1.32 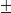 0.04
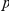	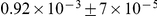	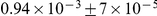	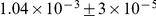	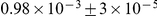

Coefficients of Equations 5 and 6 (fit with 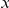 and 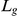) and of Equations 7 and 8 (fit with 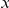, 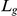, 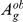 and 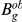) determined by fitting the data 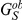 and 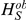 of *S. cerevisiae* genes. NA, not applicable.

We have previously seen that the GC3 content of *S. cerevisiae* genes could be considered as close to equilibrium. In the frame of the model, this observation means that the time-averaged frequencies of conversion tract initiation 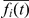 have been recently constant in *S. cerevisiae* lineage, so that 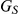 has adapted to them (see Discussion). In that case, one possibility is that not only the time-averaged frequencies 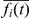, but also the frequencies 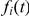 themselves could have been recently constant in *S. cerevisiae* lineage, and therefore that 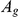 and 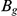, which are functions of 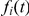, could be correctly approximated by current estimates of these frequencies. The relevance of this assumption can easily be assessed by comparing the correlations between the data 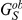 and the theoretical 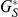 calculated with or without the approximations for 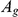 and 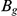.


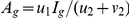
 with 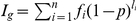. 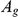 is thus proportional to the frequency 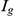 with which conversion tracts initiated at DSB sites located at distance 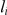 upstream of the sequence 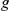 extend up to its ATG. Buhler *et al.* recently measured the amounts of ssDNA produced by the 5′ to 3′ resection of meiotic DSB ends at 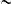 41,000 positions in *S. cerevisiae* genome [15]. The amount of ssDNA measured at a given position thus reflects the frequency with which 5′ to 3′ resection tracts, originating from DSB sites located either upstream or downstream, extend up to that position. Conversion tracts and resection tracts probably do not coincide exactly but they clearly overlap (Figure 1). I therefore hypothesized that, for a given sequence, the time-averaged frequency of conversion tract extension 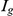 could be approximated by the current frequency of conversion tract extension, which in turn could be approximated by the current frequency of resection tract extension, estimated from ssDNA measurements. The reasoning is the same for 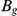 and leads to the hypothesis that 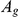 and 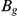 could be considered as proportional to 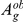 and 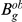, defined as the quantifications of ssDNA averaged over the 500 bp upstream of the genes start codon or over the 500 bp downstream of the genes stop codon, respectively (see Methods).

As alluded to above, these approximations of 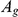 and 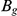 have several limitations: (i) little is known about the extension of resection tracts and how it correlates with the extension of conversion tracts, (ii) 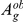 and 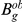 integrate the amounts of ssDNA deriving from DSBs located either upstream or downstream of the genes, whereas 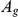 and 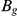 correspond to conversion tracts originating exclusively either from upstream (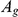) or from downstream (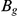) regions of the genes and (iii) the measures of ssDNA appear noisy as the Pearson's correlation coefficient between the values determined by Buhler *et al.* and by another study [14] analyzing the same strain with the same microarrays and protocols is 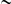 0.62 (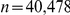).

Replacing 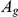 and 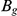 by 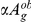 and 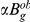 in Equations 5 and 6 gives 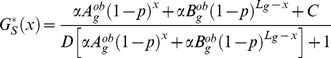
(7)

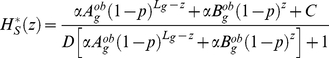
(8)


Estimates of 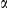, 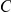, 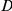 and 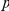 were determined by fitting the observed GC3 contents 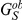 and 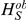 to Equations 7 and 8 (Table 1). The values of 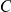 and 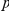 were close to the ones previously found. 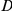 was found equal to 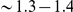, which corresponds to 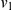 equal to 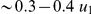, 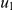 and 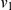 being the AT to GC and GC to AT substitution rates linked to recombination-dependent processes, respectively. Importantly, the Pearson's correlation coefficients between 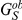 or 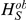 and the new theoretical values 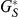 or 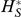 that can be calculated as functions of 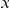, 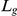, 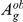 and 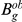 using Equations 7 and 8 were equal to 0.44 in both cases (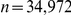). Approximating 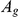 and 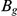 by 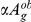 and 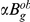 thus results in a significant increase in the correlations between 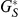 and 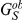 and between 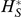 and 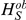, which suggests that 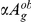 and 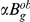 are relevant approximations of 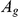 and 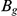, and therefore that the frequencies of conversion tract initiation 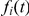 have been recently constant in *S. cerevisiae* lineage. The curves corresponding to the mean values of 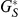 and 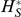 obtained with Equations 7 and 8 and averaged over all genes are shown in Figure 3.

The slopes of the curves representing 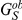 and 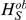 also behaved as expected from the model. Intuitively we expect the curve 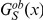 (respectively, 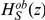) to be almost flat near 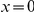 (respectively, near 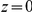) for genes with low 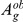 (respectively, low 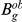) and to present a steeper slope for genes with high 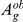 (respectively, high 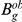). These intuitions can be formalized by calculating the derivatives of 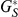 and 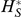 with respect to 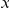 and 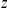, respectively 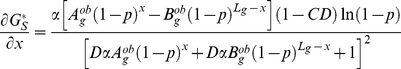
(9)

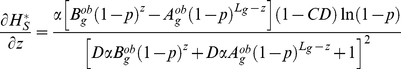
(10)


At the positions 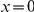 and 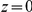, we thus have 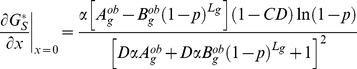
(11)

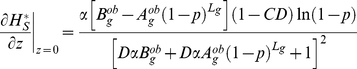
(12)


At the denominator, 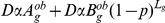 and 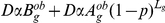 can be neglected compared to 1 as a first approximation. At the numerator, 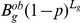 and 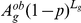 become negligible compared to 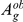 and 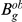, respectively, for large values of 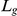. The initial slopes of 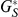 and 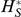 are thus roughly proportional to 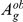 and 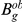, respectively, for long genes.

**Figure 4:**
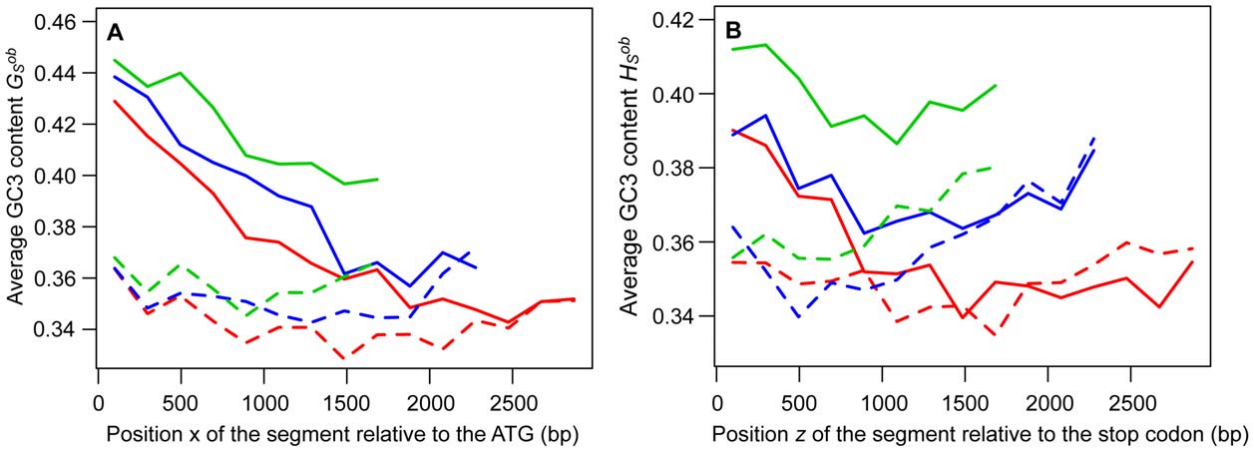
The initial slopes of 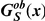 and 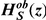 are correlated with the values of 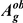 and 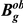 for *S. cerevisiae* genes. The mean GC3 contents 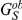 (A) or 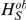 (B) of 66-codon segments are plotted as a function of the positions 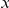 or 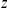 relative to the ATG or to the stop codon, respectively, on which the segments are centered. 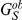 and 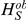 are averaged over classes of genes sorted by their lengths and their values of 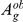 and 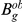. The green curves correspond to the genes containing 9 to 11 66-codon segments, the blue curves, to the genes containing 12 to 14 segments and the red curves, to the genes containing at least 15 segments. For each set of genes, the solid curves represent the genes with high values of either 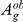 or 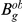, and the dashed curves represent the genes with low values of either 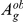 or 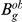.

Three sets of long genes were analyzed for comparison with this theoretical result. For each set, the average GC3 contents 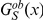 and 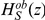 of 66-codon segments centered on position 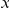 from the ATG or on position 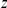 from the stop codon, respectively, were calculated for two groups of genes with either high or low 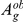 or 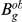 (whose 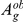 or 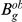 fall either within the first or the last quartile). As shown in Figure 4, the initial slope of 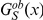 and 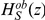 was indeed higher for genes with higher 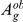 and 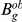, respectively.

The relationship between GC3 content, 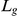, 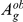 and 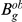 can also be expressed at the level of whole genes by integrating Equation 7 to determine 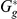, the gene equilibrium GC3 content 
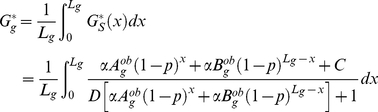
(13)


According to the sign of 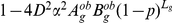, we get either 
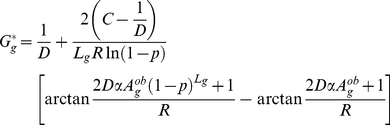
(14)with 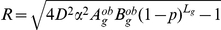, or 
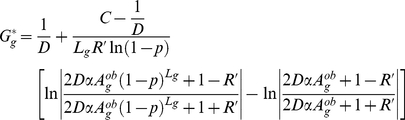
(15)with 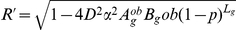.

**Figure 5:**
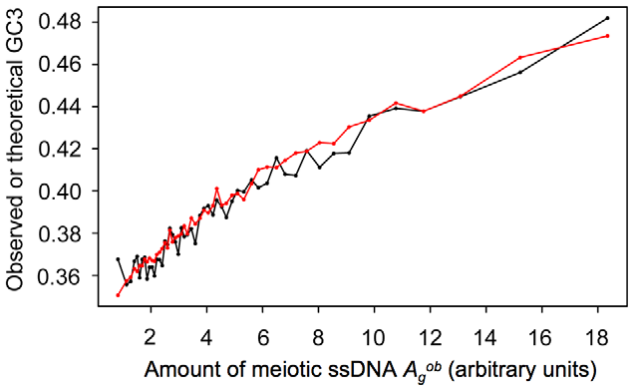
Variations of the GC3 contents of *S. cerevisiae* genes as a function of the amount of ssDNA measured during meiosis over the 500 bp upstream of their start codons (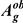). The mean observed GC3 content 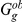 (black curve) and the mean theoretical GC3 content 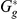 calculated as a function of 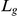, 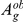 and 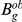 (red curve) were averaged over groups of 100 genes binned by their values of 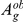, and plotted as a function of 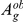 (in arbitrary units).

Figure 5 shows the mean values of 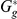 and of 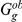 (the observed GC3 content of genes) averaged over groups of 100 genes binned by their values of 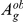, *i.e.* by the average amount of ssDNA measured by Buhler *et al.* during meiosis over the 500 bp upstream of their start codons [15]. 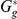 was calculated as a function of 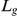, 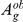 and 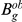 (Equations 14 and 15) using the estimates of 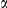, 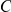, 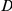 and 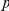 previously determined by fitting the data 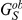 (Table 1). The Pearson's correlation coefficient between 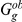 and 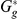 was equal to 0.58 (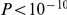, 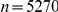).

Several observations thus argue that the model provides a relevant description of the variations in the average GC3 content of *S. cerevisiae* genes: (i) these variations present the expected shape with higher GC3 content at the ends of the genes, (ii) they can be fitted by curves described by Equations 5 and 6, (iii) the theoretical values 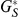 and 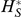 calculated from Equations 7 and 8 as functions of 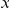, 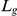, and the approximations 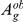 and 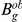 are highly correlated with the observed data 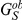 and 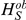, and (iv) the estimates of parameters relative to meiotic recombination are consistent with experimental observations (the estimates of 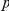 coincide with its experimentally determined value [9], and the estimates of 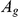 are higher than the estimates of 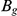).

Since in that case variations in the average GC3 content seem to reflect GC-biased gene conversion linked to meiotic recombination, it is conceivable that the analysis of GC content could provide some information on the mechanisms of meiotic recombination in organisms where it is less studied than in *S. cerevisiae*. I then sought to analyze the genome of yeasts related *S. cerevisiae* (belonging to the same class of hemiascomycetes), with the idea that some features of meiotic recombination required for the application of the model will be present in these yeasts as they are in *S. cerevisiae*.

### Analysis of *Candida* coding sequences

Species belonging to the *Candida* genus and their relatives are particularly interesting in that they offer a diversity of sexual lifestyles (for a review see [16]). Thus, *C. lusitaniae*, *C. guilliermondii*, and *Debaryomyces hansenii* are clearly sexual. Interestingly, *C. lusitaniae*, although it lacks many key meiotic components including the recombinase Dmc1, undergoes Spo11-mediated meiotic recombination at frequencies similar to that of other sexual fungi [17]. *Lodderomyces elongisporus* has been described as a diploid, homothallic species capable of sporulation, but a sexual cycle has never been formally demonstrated [16]. *C. parapsilosis* and *C. tropicalis* have many of the genes required for meiosis and mating, but sex has never been observed in these species [16]. Finally, meiosis has never been observed in *C. albicans* but this yeast presents a parasexual cycle in which mating of diploid cells to form tetraploid cells is followed by random chromosome loss to generate diploid progeny cells. Recombination in *C. albicans* undergoing the parasexual pathway is Spo11-dependent but less frequent than that expected from a classical meiotic pathway [18]. This latter observation is consistent with population studies of clinical isolates of *C. albicans* strains indicating limited genetic exchange for these yeasts in their natural environment [19].

**Figure 6:**
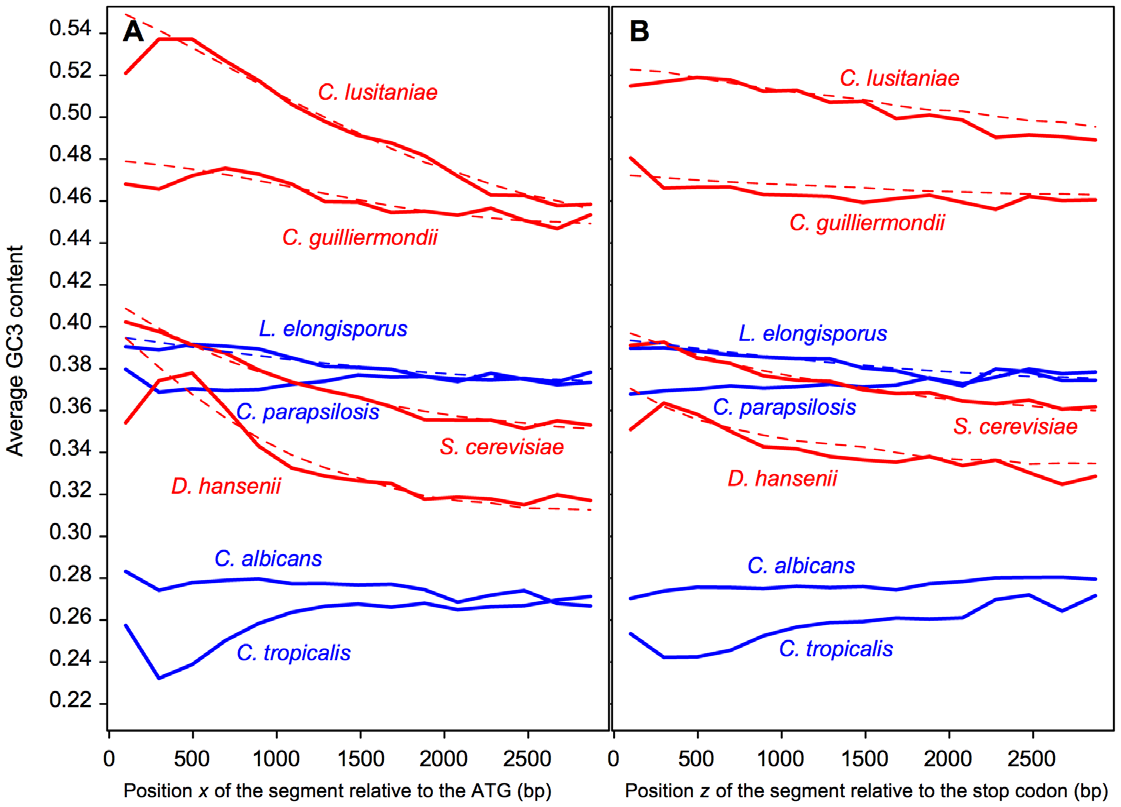
Variations of GC3 content in *Candida* protein-coding sequences. The mean GC3 contents 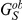 (A) or 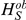 (B) of 66-codon segments (thick, solid curves) are plotted as a function of the positions 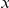 or 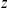 relative to the ATG or to the stop codon, respectively, on which the segments are centered. For each position 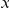 or 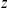, 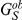 or 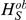 are averaged over all the genes of a given species. The thin, dashed lines represent the mean theoretical values 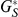 or 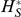. The values of 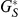 (or 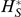) were calculated for all segments as functions of 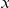 (or 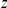) and 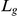, using Equation 5 (or Equation 6) and the estimates of 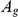, 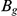, 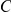, 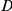 and 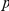 given in Table 2. The values of 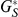 or 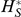 were then averaged over segments with the same position 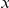 or 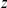. Red and blue curves correspond to species with an established sexual cycle, and to species in which sex has never been observed, respectively.

If we presume that species with established sexual cycles also exhibit GC-biased gene conversion linked to meiotic recombination, and meiotic DSB sites preferentially located outside of protein-coding sequences, then we expect that the average GC3 content of their gene segments (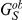 and 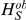) will follow the same type of curves than those observed for *S. cerevisiae*. As shown in Figure 6, the curves corresponding to 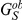 and 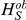 for the species *C. lusitaniae*, *C. guilliermondii*, and *D. hansenii* display a decreasing trend for increasing 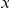 and 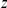, and are comparable to those of *S. cerevisiae*. In some cases however, the first gene segment exhibits an aberrant behavior (see in particular the curves representing 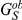 for *C. lusitaniae* and *D. hansenii* in Figure 6A). These abnormalities could be due to an erroneous determination of the translation start site or to constraints affecting specifically the GC3 content of the end segments.

Since no measurements of local meiotic DSB frequencies are available for these species, the observed data 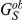 (excluding the first gene segment for *C. lusitaniae* and *D. hansenii*, and the first two gene segments for *C. guilliermondii*) were fitted with Equation 5, considering uniform 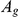 and 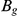 values for all genes. Estimates of the parameters 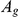, 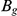, 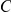, 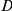 and 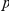 are shown in Table 2. The values obtained by fitting *S. cerevisiae* 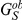 to the exclusion of the data corresponding to the first gene segment are also shown for a comparison with previous estimates (Table 1, second column) and indicate that in this case the removal of these data has little influence on the results.

**Table 2:** Equation coefficients for the genes of *Candida* and related species.

	*C. lusitaniae*	*C. guilliermondii*	*L. elongisporus*	*D. hansenii*	*S. cerevisiae*
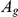	1.9 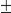 0.4	7 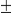 4	0.2 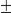 0.1	0.7 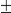 0.1	0.13 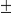 0.04
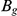	0.6 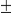 0.1	1.4 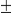 0.5	0.2 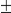 0.1	0.34 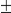 0.05	0.09 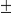 0.03
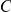	0.39 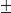 0.01	0.429 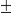 0.006	0.349 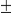 0.006	0.277 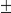 0.003	0.322 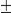 0.003
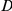	1.67 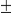 0.04	2.07 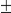 0.02	2.1 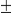 0.2	2.11 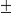 0.07	1.0 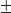 0.4
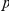	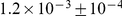	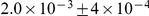	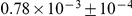	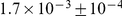	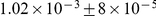

Coefficients of Equation 5 determined by fitting 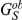 of *C. lusitaniae*, *C. guilliermondii*, *L. elongisporus*, *D. hansenii* and *S. cerevisiae* genes to the exclusion of the data corresponding to the first gene segment for all species, except for *C. guilliermondii* for which the data corresponding to the first two gene segments were excluded from the analysis.

Two main observations can be gathered from Table 2. First, the estimates of 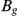 are systematically lower than the estimates of 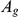, which, in the frame of the model, indicates that the frequency of meiotic DSBs is higher in the upstream regions of the genes than in their downstream regions, in agreement with what is observed in *S. cerevisiae*. Second, estimates of 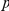 are rather homogeneous and range between 0.001 and 0.002, close to the estimate obtained for *S. cerevisiae*.

Let's consider now the species in which sex has never been observed. The curves corresponding to 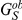 and 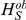 for *L. elongisporus* exhibit a slight but significant decreasing trend and could be fitted by Equation 5 (the estimates of the parameters are given in Table 2). In the frame of the model, these observations suggest either that *L. elongisporus* is sexual but undergo meiosis at low frequency, or that it belongs to a recent asexual lineage so that the sexuality of its ancestors is still reflected in the variations of its GC3 content. In contrast, the 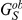 and 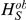 curves for *C. parapsilosis*, *C. albicans*, and *C. tropicalis* are either flat or with an increasing trend and cannot be described by the model. This could be interpreted as an evidence for their belonging to ancient asexual lineages, although we cannot rule out that the model would not apply to them for other reasons, because they would lack GC-biased gene conversion linked to meiotic recombination, or because protein-coding sequences would experience meiotic DSBs with the same frequency as the rest of the genome. The Spo11-dependent recombination of *C. albicans* linked to its parasexual pathway has not been extensively characterized. Our analysis suggests that either it operates at low frequency or that it is not accompanied by GC-biased gene conversion or that the initiating DSBs are not excluded from gene sequences.

In summary, the sequence analysis of yeast species belonging to or close to the *Candida* genus suggests that meiotic recombination in the sexual species is similar to that of *S. cerevisiae*, with associated GC-biased gene conversion, exclusion of DSBs from protein-coding sequences, preferential localization of DSBs in the gene upstream regions, and similar probability of conversion tracts to stop at each base pair (hence similar conversion tract length). Regarding the species in which sex has never been observed, either the model was consistent with low frequency of meiotic events or could not fit the data, which could be interpreted as an evidence for their ancient asexuality having erased the genomic traces of their ancestors' sexual life. The observations of GC3 variations in these yeasts were thus consistent with the model's predictions given their sexual lifestyles.

### Analysis of the coding sequences of higher eukaryotes

The existence of GC-biased gene conversion has been inferred in mammals and in birds, based on strong correlations at megabase (Mb) scales between crossover rates and current or equilibrium GC contents [20]–[22], so the first condition of application of the model should be met for these organisms. Regarding the location of meiotic DSBs, human historical hotspots seem to locate preferentially outside genes [23] but the few mouse hotspots analyzed at a sub-kb scale for crossover activity were found in regions containing both exons and introns as well as in coding deserts [24], so it is not clear to what extent meiotic DSBs are excluded from coding regions in the genomes of higher eukaryotes. A major complication arising in the analysis of the GC content of higher eukaryotes lies in the existence of so-called CpG islands, which are regions of DNA with a high GC content and a high frequency of CpG dinucleotides. CpG islands are frequently associated to promoter regions, extending through 5′-flanking DNA, exons and introns, but are also found in the 3′ end of some genes [25]. To simplify the interpretation of the results, all exons overlapping a CpG island were removed from the analysis (see Methods).

**Figure 7:**
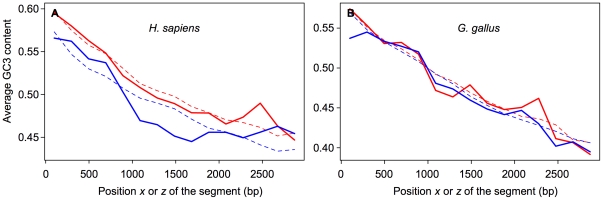
Variations of GC3 content in human and in chicken protein-coding sequences. The mean GC3 contents 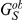 of 66-codon segments are plotted as a function either of the segment position 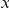 relative to the ATG (red solid line) or of the segment position 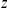 relative to the stop codon (blue solid line). For each position 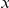 or 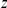, 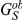 or 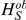 are averaged over all the first coding exons or over all the last coding exons, respectively, of human (A) or chicken (B) protein-coding sequences. The dashed lines represent the mean theoretical values 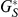 (red) or 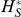 (blue). The values of 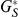 (or 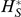) were calculated as functions of 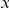 (or 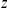) and 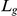, using Equations 5 (or 6) with the estimates of 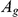, 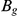, 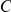, 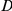 and 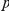 given in Table 3.

The GC3 content of human coding sequences was analyzed by considering all the single-exon genes and either the first coding exon of intron-containing genes for 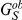, or the last coding exon of intron-containing genes for 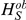. As shown in Figure 7A, the average GC3 content of the exon segments exhibited a decreasing trend with increasing distance either from the ATG or from the stop codon. The estimates of the parameters obtained by fitting 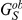 with Equation 5 and 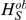 with Equation 6 are given in Table 3. The Pearson's correlation coefficients either between 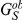 and the theoretical GC3 content 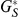 calculated from Equation 5 or between 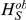 and the theoretical GC3 content 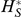 calculated from Equation 6 are equal to 0.28 (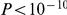, 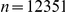) and to 0.30 (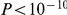, 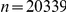), respectively.

**Table 3:** Equation coefficients for the genes of *H. sapiens* and of *G. gallus.*

	*H. sapiens*		*G. gallus*	
	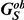	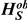	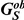	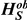
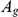	0.4 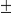 0.1	0.31 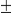 0.06	0.4 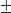 0.1	0.5 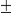 0.2
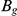	0.35 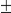 0.09	0.41 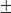 0.08	0.4 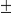 0.1	0.4 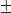 0.2
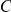	0.37 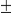 0.02	0.35 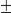 0.01	0.27 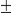 0.04	0.21 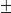 0.06
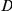	1.0 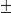 0.2	1.0 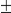 0.1	1.0 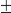 0.2	1.0 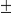 0.2
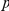	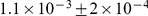	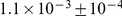	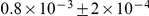	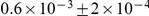

Coefficients of Equations 5 and 6 determined by fitting 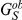 and 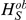 for *H. sapiens* and *G. gallus* genes. All the data 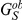 and 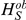 were taken into account, except in the case of *G. gallus* 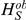 where the coefficients of Equation 6 were estimated by fitting 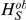 to the exclusion of the data corresponding to the first segment of the exons.

Ideally, one would like to be able to test the relevance of the model for human sequences by comparing the variations of GC3 content in loci with different meiotic DSB frequencies. However, the correlation between the equilibrium GC contents and estimates of the historical crossover rates is strongest at the 10-Mb scale but decreases with decreasing scales to become very weak below 200 kb [20], that is at scales much larger than the scale of gene length. Besides, the non-recombining Y chromosome harbors too few genes to allow for a valuable comparison with the other chromosomes.

The same analysis was performed on the coding sequences of the chicken (*Gallus gallus*). The GC3 content of exon segments also decreases regularly as a function of their distance from the ATG or from the stop codon (Figure 7B). The estimates of the parameters obtained by fitting 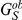 and 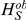 with Equations 5 and 6 are given in Table 3. The Pearson's correlation coefficients between 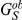 and 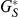 and between 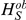 and 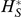 are equal to 0.32 (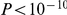, 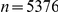) and to 0.23 (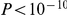, 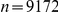), respectively.

The coding sequences of both *H. sapiens* and *G. gallus* thus exhibit variations in their GC3 contents that are consistent with the model. The estimates of the coefficients 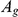 and 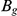 are quite close in both cases, suggesting that the frequencies of conversion tract initiation are comparable in the 5′ and in the 3′ ends of the genes. Finally, the estimates of 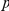, the probability of conversion tracts to stop at each base pair, are similar to those obtained from the previous analyses of the yeast genomes.

## Discussion

A model was devised for the evolution of the GC content of sequences submitted to meiotic GC-biased gene conversion but devoid of meiotic DSB sites. An equation (Equation 5) was derived describing the equilibrium GC content of these sequences according to the model. Although this equation entails many simplifying assumptions, it seems to capture the average variations of GC3 content of protein-coding sequences in several eukaryotic genomes.

### A potentially universal pattern of GC variations in the coding sequences of eukaryotic genes

The relevance of the model was best tested with *S. cerevisiae*, for which a wealth of quantitative data is available regarding recombination. The goodness of fit between the theoretical and the observed GC3 contents, and the consistency between experimental data and estimates of meiotic recombination parameters that can be derived from the model, are strong arguments in favor of its pertinence. Analysis of *Candida* and related species provided more support to the model as the differences observed in the variations of GC3 content between species with an established sexual cycle and species in which sex has never been observed, are consistent with its expectations. Finally, the theoretical and the observed GC3 contents for the human and the chicken sequences also showed a good agreement, consistent with the presumed existence of GC-biased gene conversion in these species. It has to be noted that in the case of higher eukaryotes, genomic sequences of asexual species are not available, and therefore we lack negative controls for the model. We cannot rule out the possibility that the criteria for the elimination of sequences containing CpG islands might not be stringent enough, or that other phenomena, linked to transcription or to translation, could induce the observed variations in GC3 content.

However, in spite of these caveats, the diversity of the species for which we have observed a good correlation between the model and genomic data suggests that the model could apply to many eukaryotes, which would imply the generality of two features of meiotic recombination required for its application, namely the existence of GC-biased gene conversion and the quasi-exclusion of meiotic DSBs from coding sequences. In these cases, analysis of GC variation could provide some information on the parameters of meiotic recombination, like the mean length of conversion tracts, and capture some characteristics of the history of meiotic recombination (hence sexuality) of a given lineage.

In a given organism to which the model applies, the magnitude of GC-biased gene conversion influence on GC3 content can be deduced directly from Equation 5. For example in *S. cerevisiae*, in the absence of meiotic recombination (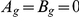), we have 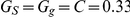. As shown in Figure 5, the highest GC3 content 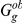 (observed for the genes with the highest 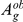) corresponds to 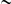 0.48. This indicates that GC-biased gene conversion should be responsible for 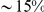 of GC bases at the third codon position in these sequences, *i.e.* for a 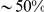 increase in GC3 content compared to the basal level of 0.33.

As could be expected, the GC1 and GC2 contents of *S. cerevisiae* coding sequences (corresponding to the GC contents of the first and the second codon positions, respectively) exhibit the same decreasing trends as GC3 with increasing distance from the translation start or stop sites, although with a smaller amplitude (data not shown). Such an influence on GC1 and GC2 contents most probably translates into an influence on amino-acid sequences. In eukaryotic genomes, GC-biased gene conversion is thus expected to bring about additional constraints acting more specifically on protein segments encoded by exon terminal sequences. Ideally these constraints should be taken into account when modelizing gene evolution.

### Analysis of S. cerevisiae

We had previously found that the correlation between recombination rate and GC content in *S. cerevisiae* was higher than the correlation between recombination rate and the equilibrium GC content GC*, which suggested that the correlation between recombination rate and GC content was mostly due to a causal influence of the GC content on the recombination rate [26]. These previous results can be explained by the fact that recombination has only a weak influence on sequence evolution in *S. cerevisiae* lineage on time scales corresponding to the time lapse between now and the time of divergence between *S. cerevisiae* and *S. paradoxus* lineages. Both Noor and I found indeed a null or negative correlation between recombination rate and non-coding or coding sequence divergence between *S. cerevisiae* and *S. paradoxus* ([27], data not shown). The weak effect of meiotic recombination on the evolution of GC content in *S. cerevisiae* lineage, combined with the short length scale (a few kbs) over which it operates (which reduces the number of observed substitutions for the determination of the equilibrium GC content and increases its variability), makes it difficult to detect the influence of recombination on GC content through correlation analyses.

A last note concerns the stability of meiotic recombination rates in *S. cerevisiae* lineage. The facts that GC3 is close to equilibrium and that 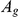 and 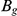 can be approximated by the current measurements 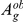 and 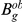 imply that the local frequencies of meiotic DSBs have been lately stable in *S. cerevisiae* lineage. A similar conclusion was recently reached by Tsai *et al.*, who observed a significant overlap between the recombination hotspots of *S. paradoxus* and *S. cerevisiae* on chromosome III [28]. The stability of DBS sites in *S. cerevisiae* lineage thus stands in contrast to the lability of DSB sites in the human lineage, in which recombination hotspots are changing at a fast step due to the rapid evolution of the DNA-binding domain of PRDM9, the histone methylase responsible for determining DSB sites [29], [30]. In yeast, a histone H3 K4 methylase, Set1, also marks DSB sites, but Set1 has no DNA-binding domain, and does not determine DSB sites [31]. DSB sites in yeast are thus likely to be determined by more complex (and more stable) parameters than consensus sequences, which remain so far unknown.

### DNA sequences as low-frequency filters of the variations of local DSB frequencies

More theoretical comments can be made upon Equation 1 describing the evolution of a sequence segment GC content (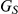). For a given segment at position 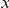, Equation 1 can be written 
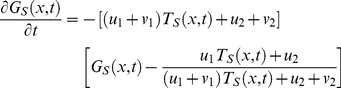
(16)


Considering 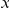 fixed and 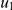, 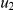, 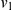, 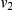 and 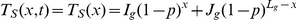 constant over time, Equation 16 corresponds to the general form 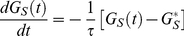
(17)describing the variations of a quantity 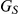 that relaxes exponentially from an initial value 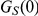 towards an equilibrium value 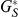 (given by Equation 4) with a relaxation time 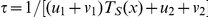. The solution of Equation 17 is 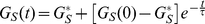
(18)


Equation 17 is of a general type that describes the behavior of many physical variables. In particular, it is exemplified by Newton's law of coolness, which describes the temperature changes of a body from an initial temperature to the equilibrium (ambient) temperature as it cools down or warms up.

Even more generally, for a given segment at position 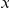, Equation 16 is an example of a differential equation characterizing a first order linear time-invariant system with the generic form 
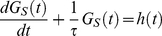
(19)with 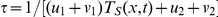 and 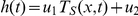. Let's recall that 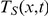 is equal to 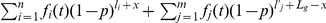 (Equation 2) with 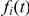 being the frequency of conversion tract initiation at each DSB site 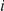. 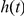 can be regarded as the system input, to which 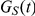 is the response, or system output.

In our model, we have considered the case where 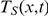 could be approximated by a function 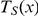 independent of time. Both 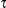 and 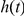 have therefore been considered constant over time, and Equation 19 in that case corresponds to Equation 17 as it describes the behavior of a system relaxing towards the equilibrium value 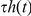.

Let's consider now the case where the values of 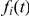 change with time. In at least some organisms like *S. cerevisiae*, there is little correlation between recombination rate and sequence divergence (see the discussion above), which indicates that 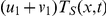 can be neglected compared to 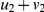. In that case, 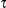 can still be considered as a constant, equal to 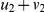. In contrast, 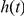 now changes with time.

Let's suppose that the frequencies of conversion tract initiation 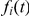 suddenly change at a given locus (for example, a new recombination hotspot appears), so that 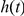 goes rapidly from 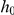 to 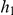. The GC content 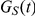 responds to this change by gradually relaxing to the new equilibrium value 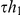 with the relaxation time 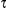. If 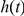 then remains equal to 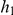 for a period representing several times the value of 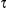, then 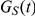 will come closer and closer to its equilibrium value 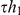. When 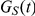 will have almost reached equilibrium, it will be highly correlated with estimates of 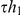, which can be obtained by a punctual measurement of local DSB frequencies at any time during this period.

In contrast, if the frequencies of conversion tract initiation 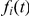 keep changing at a fast pace compared to 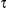 (if recombination hotspots arise and disappear quickly), 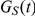 will never have the time to come close to a new equilibrium value 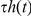 as it will only have time to make a small change towards 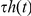 before 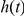 changes again. However, if 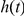 does not exhibit additional low frequency changes, the small changes in 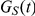 induced by the high frequency changes of 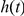 will average out. 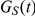 will then come close to an equilibrium value 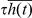, with 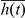 corresponding to a mean value of 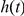 averaged over a period of time relatively large compared with the period of 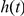 variations.


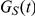
 can thus come close to equilibrium in two quite different cases, either when 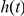 remains constant or when it exhibits rapid changes compared to 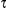. However, in this latter case, the equilibrium value 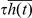 of 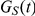 cannot be estimated by a punctual measurement since it represents a time average. The observations that the GC3 contents of *S. cerevisiae* genes are close to equilibrium and that the equilibrium values can be estimated using current measurements of local DSB frequencies therefore indicate that meiotic recombination rates have been lately stable in *S. cerevisiae* lineage.

We have just seen that changes in 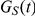 induced by high frequency changes of 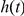 average out, so that 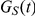 can only reflect low frequency changes of 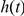. In signal processing this behavior characterizes low-pass filters, which pass low-frequency signals but reduce the amplitude of signals with higher frequencies. DNA sequences submitted to GC-biased gene conversion can therefore be considered as low-pass filters transforming temporal variations in meiotic DSB frequency into temporal variations in GC content.

Let's finally derive an approximation of 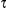 for *S. cerevisiae*. Let 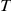 be the time elapsed since the divergence between *S. cerevisiae* and *S. paradoxus* lineages. The mean AT to GC and GC to AT substitution rates (respectively, 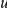 and 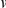) in *S. cerevisiae* lineage, were calculated for the third codon position by averaging over all genes (see Methods), and were found equal to 0.108 and 0.163, respectively. 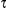 can then be approximated as 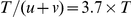. This is only an approximation since (i) the effects of GC-biased gene conversion are neglected, and (ii) no attempt is made to correct for multiple substitutions.

The fact that the GC3 contents of *S. cerevisiae* genes are close to equilibrium suggests that the meiotic recombination rates (along with the other parameters 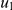, 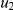, 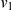 and 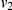) have been stable in *S. cerevisiae* for several periods of time 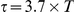. In that case, we expect that the GC3 contents 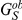 in species related to *S. cerevisiae*, like *S. paradoxus* and *S. mikatae*, will also be highly correlated to the equilibrium values 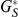 calculated using Equation 7 as a function of the gene lengths 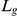 and of the values 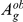 and 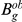 measured for the corresponding genes in *S. cerevisiae*. Indeed, even if the local frequencies of conversion tract initiation have changed in *S. paradoxus* and *S. mikatae* lineages since their divergence from the lineage of *S. cerevisiae*, their 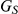 should not have had the time to adapt to these new values. In agreement with these expectations, the Pearson's correlation coefficients between 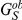 and 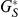 were found equal to 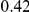 (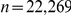) and 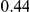 (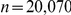) for *S. paradoxus* and *S. mikatae*, respectively.

## Methods

### Genomic data

The sequences of *S. cerevisiae* strain S288C, *S. paradoxus* and *S. mikatae* were downloaded from the Broad Institute web pages (http://www.broad.mit.edu/annotation/fungi/comp_yeasts/downloads.html; in correspondence with supplemental information in [32]). I analyzed 5500 ORFs of *S. cerevisiae* without intron, annotated as verified or uncharacterized in the Saccharomyces Genome Database (http://www.yeastgenome.org/).

The sequences of species belonging to the *Candida* genus and their relatives were downloaded from the Broad Institute web pages (http://www.broadinstitute.org/annotation/genome/candida_albicans/MultiDownloads.html; in correspondence with supplemental information in [16]). For each species, I analyzed all the sequences whose lengths were identical in the genus_species_n_transcripts.fasta and in the genus_species_n_genes.fasta files (indicative of the absence of introns). Sequences that either did not start with an ATG, or did not end with a stop codon, or whose lengths were not a multiple of 3 were discarded.

Regarding the human and chicken genomes, the sequences of single-exon genes and of the first and the last coding exons of intron-containing genes were retrieved from GenBank files downloaded from the NCBI webpage (ftp://ftp.ncbi.nih.gov/genomes/). Only autosomal chromosomes were analyzed. When relevant, sequences were checked for the presence of an initiating or of a stop codon and for an integral number of codons. When several sequences had the same GeneID reference, only the first one was taken into account. Sequences with undetermined bases were discarded. For the identification of CpG islands, I followed the method described in [25]. For each exon, a moving average value of the GC content and of the ratio observed/expected CpG (CpG[o/e]) was calculated, using a 100 bp window, for each base of the sequence starting 250 bp upstream of the exon start and ending 250 bp downstream of the exon end. Sequences were considered to contain a CpG island if, over a given stretch of 250 bp, at least 200 bp were such that their moving average values of GC and of CpG[o/e] were greater than 0.5 and 0.6, respectively. All sequences containing a CpG island were removed from the analysis.

### Substitution analyses

Substitution analyses were required to estimate (i) the equilibrium GC3 content of *S. cerevisiae* genes (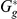) and (ii) 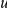 and 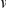, the mean AT to GC and GC to AT substitution rates, respectively, in *S. cerevisiae* lineage for the third codon position of coding sequences. The substitution analyses involved a comparison between the sequences of *S. cerevisiae* and *S. paradoxus* with *S. mikatae* as an outgroup. All the open reading frames (ORFs) with unambiguous correspondence in *S. cerevisiae*, *S. paradoxus* and *S. mikatae* (listed by Kellis and collaborators in the web page ftp://ftp-genome.wi.mit.edu/pub/annotation/fungi/comp_yeasts/S1b.ORFs/listing.txt) were selected in a first step. The analysis was then restricted to 4295 ORFs annotated as verified or uncharacterized in the Saccharomyces Genome Database. Multiple sequence alignments were performed using ClustalW (downloaded from http://www.ebi.ac.uk/Tools/clustalw/).

The substitutions in *S. cerevisiae* lineage were inferred by comparison with *S. paradoxus* sequences using parsimony on informative sites, with *S. mikatae* as an outgroup to infer the ancestral nucleotide sequences. The sites where *S. mikatae* sequences differed from the sequences of *S. cerevisiae* and *S. paradoxus* were disregarded. No attempt was made to correct for multiple substitutions. The mean substitution rates 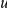 and 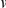 averaged over all genes for the third codon position in *S. cerevisiae* lineage were estimated by dividing the number of inferred substitutions by the number of inferred, potentially mutable, ancestral sites. The equilibrium GC3 content of individual *S. cerevisiae* genes was calculated using the model of Sueoka [33], as the ratio 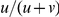.

### Estimation of the frequencies of meiotic resection tract extension in *S. cerevisiae*

Estimates of the frequencies of resection tract extension were derived from the study of Buhler and collaborators [15]. In this study, ssDNA intermediates resulting from the processing of meiotic DSBs were detected by microarray hybridization. For a given gene, I took as an estimate of the frequency with which resection tracts reach either the ATG or the stop codon, respectively, the average of the measured values for DNA probes with midpoints localized either in the 500 bp upstream of the ATG or in the 500 bp downstream of the stop codon (average ratios of background-normalized fluorescence in *dmc1* mutants).

### Numerical analyses and statistics

Data sets were produced and analyzed using custom Python scripts (http://www.python.org). All statistical analyses were performed in R (http://www.r-project.org) [34]. In particular, nonlinear regression analysis was performed using the nls() function. In some cases (mentioned in the text), the data corresponding to the first (gene or exon) segment had to be removed in order for the fit to converge.
